# Two new species of *Annulatascaceae* (*Annulatascales*, *Sordariomycetes*) from Dongting Lake

**DOI:** 10.3897/mycokeys.136.188740

**Published:** 2026-07-15

**Authors:** Zhi-Yang Wang, Vinodhini Thiyagaraja, Shu-Cheng He, Nuwan Kularathnage, Ling-Ling Yuan, Ratchadawan Cheewangkoon, Kevin D. Hyde, Qi Zhao

**Affiliations:** 1 State Key Laboratory of Phytochemistry and Natural Medicines, Kunming Institute of Botany, Chinese Academy of Sciences, Kunming, Yunnan 650201, China Center of Excellence in Fungal Research, Mae Fah Luang University Chiang Rai Thailand; 2 Faculty of Agriculture, Chiang Mai University, Chiang Mai 50200, Thailand Kunming Institute of Botany, Chinese Academy of Sciences Kunming China; 3 Center of Excellence in Fungal Research, Mae Fah Luang University, Chiang Rai 57100, Thailand Faculty of Agriculture, Chiang Mai University Chiang Mai Thailand

**Keywords:** Freshwater fungi, morphology, phylogeny, taxonomy, two new species

## Abstract

Freshwater fungi in *Annulatascaceae* represent a morphologically diverse but taxonomically challenging group, with several genera remaining poorly defined due to morphological convergence and a lack of molecular data. In this study, two novel species, *Annulatascus dongtingensis***sp. nov**. and *Fusoidigranularius dongtingensis***sp. nov**., are introduced from Dongting Lake in the mid-lower Yangtze River basin, China. Among them, *F. dongtingensis* broadens the known morphological variation of the genus by exhibiting a vertically oriented ostiolar neck. Maximum likelihood and Bayesian analyses of ITS, LSU, and SSU sequences showed that both species represent distinct lineages. *Annulatascus dongtingensis* formed a sister clade to *A. hongkongensis* and *A. thailandensis*, whereas *Fusoidigranularius dongtingensis* grouped with its type species, *F. nilensis*.

## Introduction

Freshwater fungi are ecologically important and highly diverse, but they remain taxonomically challenging due to the frequent occurrence of cryptic species adapted to local environmental conditions (Shearer et al. 2009; Zhang et al. 2017). Although they were traditionally classified based on shared morphological characteristics, phylogenetic analyses have demonstrated that they are polyphyletic, including *Annulatascaceae*-like taxa, especially the genus *Annulatascus* (Abdel-Wahab et al. 2011; Zelski 2015). Subsequent taxonomic revisions, including the establishment of *Atractosporales*, have not fully resolved their relationships due to limited molecular data, resulting in unstable classifications and frequent reassignments (Zhang et al. 2017; Dong et al. 2021). In China, studies on freshwater fungi in streams and lakes have become relatively common compared to those in the past, with data from the Hong Kong Special Administrative Region (Tsui et al. 2000; Ho et al. 2002), Yunnan Province (Cai et al. 2002; Luo et al. 2018), and Xizang Autonomous Region (Xu et al. 2025). In this study, taxa in *Annulatascaceae* that have long been recognized as one of the morphologically distinctive but systematically unstable families were investigated (Maharachchikumbura et al. 2015; Zhang et al. 2017; Hyde et al. 2020; Dong et al. 2021). Members of *Annulatascaceae* typically possess cylindrical, thin-walled asci bearing a large, J- apical ring and ascospores usually with appendages or sheaths (Hyde 1992; Wong et al. 1998; Maharachchikumbura et al. 2015). These characters once provided a stable morphological framework for classification. However, subsequent molecular studies have revealed extensive polyphyly within *Annulatascaceae* and allied taxa, resulting in major taxonomic revisions and the establishment of new orders and families to accommodate former annulatascaceous characters (Abdel-Wahab et al. 2011; Zhang et al. 2017; Luo et al. 2019; Dong et al. 2021).

*Annulatascus*, the type genus of the family, remains one of the core lineages within *Annulatascales*, yet species delimitations have repeatedly required re-evaluation (Hyde et al. 2020; Dong et al. 2021; Yang et al. 2023). Members of this genus are characterized by immersed to superficial, brown to black ascomata with long or short necks; unitunicate, cylindrical, pedicellate asci with a relatively massive, refractive apical ring; and mostly uniseriate, hyaline, fusiform ascospores, often with appendages or sheaths (Hyde 1992; Luo et al. 2015; Yang et al. 2023). Several taxa historically placed in *Annulatascus* were shown to be phylogenetically distant, leading to the reassignment of species and the erection of new genera (Dong et al. 2021). For example, *A. aquatorbae* was transferred to the newly established genus *Longivarius* based on its unevenly colored ascospores with brown central cells and subhyaline end cells. Similarly, *A. nilensis* was transferred to *Fusoidigranularius* based on its morphological features, such as immersed ascomata oriented horizontally on the substrate with a laterally growing, upward-curving neck, and 5–9(–11)-septate ascospores with an irregular granular sheath, as well as support from phylogenetic analyses (Dong et al. 2021). Despite these advances, species diversity in *Annulatascus* remains poorly understood, and further studies based on newly collected material worldwide are needed to better delimit the genus.

*Fusoidigranularius* is a recently established genus segregated from *Annulatascus* to accommodate taxa with distinct morphological and phylogenetic characteristics (Dong et al. 2021). The type species, *F. nilensis* (≡ *Annulatascus nilensis*), was initially placed in *Annulatascus* based on LSU sequence data, despite weak phylogenetic support (Abdel-Wahab et al. 2011). Subsequent studies showed that it forms a distinct lineage and differs morphologically in having horizontally oriented, immersed ascomata with laterally emerging necks and 5–9(–11)-septate ascospores with an irregular, granular sheath (Luo et al. 2019; Hyde et al. 2020; Dong et al. 2021). Although *Fusoidigranularius* has been introduced to resolve this taxonomic placement, its familial position remains uncertain, and its diversity is still poorly known, highlighting the need for further collections and phylogenetic studies (Dong et al. 2021). Continued surveys in freshwater ecosystems, especially in subtropical and tropical Asia, have consistently revealed cryptic species diversity within *Annulatascaceae* (Dong et al. 2021; Yang et al. 2023; Karimi et al. 2025).

In the present study, two novel species belonging to *Annulatascus* and *Fusoidigranularius*, respectively, are described based on fresh collections from submerged wood in Dongting Lake (Thiyagaraja et al. 2024). Integrating morphological observations with multi-locus phylogenetic analyses, their taxonomic placements are clarified, further contributing to an improved understanding of species diversity and evolutionary relationships within *Annulatascaceae*.

## Materials and methods

### Collection and morphological studies

The submerged wood specimens were collected from the shoreline of Dongting Lake in Yiyang City, Hunan Province, China, in December 2024. The specimens were placed in a zip-lock bag and returned to the laboratory for further examination. Specimens were examined following the methods outlined by Senanayake et al. (2020). Samples were observed and examined using a stereomicroscope (SteREO Discovery V12, Carl Zeiss Microscopy GmbH, Germany) and a compound microscope (Nikon ECLIPSE 80i, Nikon, Japan) connected to a Nikon DS-Ri2 digital camera (Nikon, Japan). The dimensional measurements of ascomata, peridium, hamathecium, asci, and ascospores were evaluated with Tarosoft (R) Image Frame Work version 0.9.7 (Tarosoft, Thailand). The photo plates were made using Adobe Photoshop CS6 software (Adobe Systems, San Jose, CA, USA). Single-spore isolation was performed using the direct streaking method (Senanayake et al. 2020) on fresh 4% potato dextrose agar (PDA). After germination, a single ascospore was transferred to another new PDA by a sterile needle. The cultures were developed at 25 °C in a dark incubator and checked weekly for colonies.

### DNA extraction, PCR amplification, and sequencing

Genomic DNA was extracted from mycelium scraped from colonies growing in PDA cultures for 30 days using the Trelief® Hi-Pure Plant Genomic DNA Kit (Tsingke Biotechnology Co., Ltd., China) according to the manufacturer’s instructions. The extracted DNA was stored at –20 °C for long-term storage. The internal transcribed spacer (ITS), large subunit (LSU), and small subunit (SSU) regions were amplified by PCR using genomic DNA as the template, with the primer pairs ITS4/ITS5 (White et al. 1990), LR5/LR0R (Vilgalys and Hester 1990), and NS1/NS4 (White et al. 1990). The PCR reaction was performed with a 25 μL reaction volume, which included 21 μL 2× Rapid Taq Master Mix (Vazyme P222, Nanjing Vazyme Biotech Co., China), 1 μL of each forward and reverse primer (10 μM), and 1 μL fungal genomic DNA template. Amplifications of the ITS, LSU, and SSU regions were conducted under the following conditions: pre-denaturation at 95 °C for 5 min, followed by 40 cycles of denaturation at 95 °C for 15 s, annealing at 55 °C for 15 s, elongation at 72 °C for 15 s, and a final elongation at 72 °C for 10 min. The PCR amplification products were analyzed by gel electrophoresis on 1% agarose gels stained with Gel Green. The PCR amplification products were sequenced by Shanghai Sangon Biological Engineering Technology and Service Co., Shanghai, China.

### Sequence alignment and phylogenetic analyses

The newly generated forward and reverse sequences were assembled using SeqMan Pro v. 10.0.1 (DNASTAR, Madison, USA). The concatenated sequences were subjected to a BLAST search on NCBI (https://blast.ncbi.nlm.nih.gov/Blast.cgi). Closely related taxa from representative genera of *Annulatascaceae* were downloaded from GenBank (Table 1) (Yang et al. 2023). *Ophiostoma piliferum* (AFTOL ID 910) and *O. stenoceras* (AFTOL ID 1038) were selected as the outgroup taxa following Yang et al. (2023). Sequence data were manually edited using BioEdit v.7.2.5 (developed by Tom Hall, USA) and aligned using the MAFFT v.7.110 online program (Hall 1999; Katoh and Standley 2013). Manual trimming was performed after alignment to remove ambiguous regions, including positions where insertions, deletions, or nucleotide variations were present in only a single sequence (i.e., singleton polymorphisms). The ITS, LSU, and SSU sequence data were compiled into a combined dataset using Sequence Matrix v.1.7.8 (Vaidya et al. 2011).

**Table 1.** GenBank accession numbers of the taxa used in the phylogenetic analyses in this study.

**Taxa names**	**Voucher**	**GenBank accession numbers**			**References**
		**ITS**	**LSU**	**SSU**	
*Annulatascus chiangmaiensis*	MFLUCC 17-2346 ^T^	MW286490	MW287764	**–**	Dong et al. 2021
***Annulatascus dongtingensis***	**KUNCC 25-20208 ^T^**	**PZ174008**	**PZ174012**	**PZ174016**	**This study**
***Annulatascus dongtingensis***	**KUNCC 25-20209**	**PZ174009**	**PZ174013**	**PZ174017**	**This study**
*Annulatascus hongkongensis*	HKUCC 3702 ^T^	**–**	AF132319	**–**	Ho et al. 1999
*Annulatascus nakhonensis*	MFLUCC 18-1239 ^T^	MW286505	MW287779	–	Dong et al. 2021
*Annulatascus saprophyticus*	MFLUCC 14-0035 ^T^	**–**	NG_228740	–	Luo et al. 2015
*Annulatascus saprophyticus*	KUNCC 24-18279	PV921627	PV921611	–	Unpublished
*Annulatascus saprophyticus*	KUNCC 24-17805	PV921626	PV921610	–	Unpublished
*Annulatascus songkhlaensis*	MFLUCC 18-1151 ^T^	MW286483	NG_075406	–	Dong et al. 2021
*Annulatascus thailandensis*	MFLUCC 18-1248 ^T^	NR_171953	NG_073783	NG_073526	Hyde et al. 2020
*Annulatascus tratensis*	MFLUCC 17-2055 ^T^	NR_197513	OP377977	NG_242886	Yang et al. 2023
*Annulatascus tratensis*	MFLUCC 17-2123	OP377886	OP377972	–	Yang et al. 2023
*Annulatascus velatisporus*	MFLU 16-2204	KX772399	KX772397	KX772398	Dayarathne et al. 2016
*Annulatascus velatisporus*	MFLUCC 16-1441 ^T^	KY320183	KY244031	KY244032	Dayarathne et al. 2016
*Annulusmagnus triseptatus*	MFLUCC 18-1335	MK828653	MK849799	MK828319	Luo et al. 2019
*Annulusmagnus triseptatus*	MFLUCC 17-0469	MK828655	MK849801	MK828321	Luo et al. 2019
*Annulusmagnus triseptatus*	CBS 127688	MH864680	MH876116	**–**	Vu et al. 2019
*Ascitendus aquaticus*	MR150 ^T^	–	MG813818	MG813819	Jayawardena et al. 2018
*Ascitendus austriacus*	CBS 102665 ^T^	–	NG_056942	NG_061014	Réblová and Winka 2001
*Ascitendus austriacus*	A44-28A	KU975065	AY590292	–	Unpublished
*Ascitendus austriacus*	A324-1B	–	AY590293	–	Campbell and Shearer 2004
*Fusoidigranularius nilensis*	IMI 397966 ^T^	–	HQ616536	–	Abdel-Wahab et al. 2011
***Fusoidigranularius dongtingensis***	**KUNCC 25-20200 ^T^**	**PZ174006**	**PZ174010**	**PZ174014**	**This study**
***Fusoidigranularius dongtingensis***	**KUNCC 25-20203**	**PZ174007**	**PZ174011**	**PZ174015**	**This study**
*Longicollum biappendiculatum*	PE0017-1a	KU975062	KU975071	–	Unpublished
*Longicollum biappendiculatum*	PE0017-1b	–	KU975072	–	Unpublished
*Longivarius aquatorbae*	SS 2424 ^T^	–	JN226107	JN226106	Boonyuen et al. 2012
*Ophiostoma piliferum*	AFTOL-ID 910	–	DQ470955	DQ471003	Spatafora et al. 2006
*Ophiostoma stenoceras*	AFTOL-ID 1038	–	DQ836904	DQ836897	Zhang et al. 2006
*Pseudoproboscispora caudae-suis*	A40-1a	–	AY094191	**–**	Campbell and Shearer 2004
*Pseudoproboscispora caudae-suis*	A336-2d	KU975068	AY094192	**–**	Campbell and Shearer 2004
*Submersisphaeria aquatica*	A95-1B	KU975067	AY094193	**–**	Campbell and Shearer 2004
*Submersisphaeria aquatica*	A354-1C	MW286490	AY094194	**–**	Campbell and Shearer 2004

**Notes**. The newly generated sequences are in bold, while the type strains are represented by ^T^. “–” indicates unavailable sequences. AFTOL-ID = Assembling the Fungal Tree of Life project strain number; CBS = Centraalbureau voor Schimmelcultures (Westerdijk Fungal Biodiversity Institute); HKUCC = The University of Hong Kong Culture Collection; IMI = International Mycological Institute (CABI); KUNCC = Kunming Institute of Botany Culture Collection; MFLU = Mae Fah Luang University Herbarium; MFLUCC = Mae Fah Luang University Culture Collection. A, PE, MR, and SS = isolate codes from original studies or laboratory collections not deposited in public culture collections.

**Figure 1.**
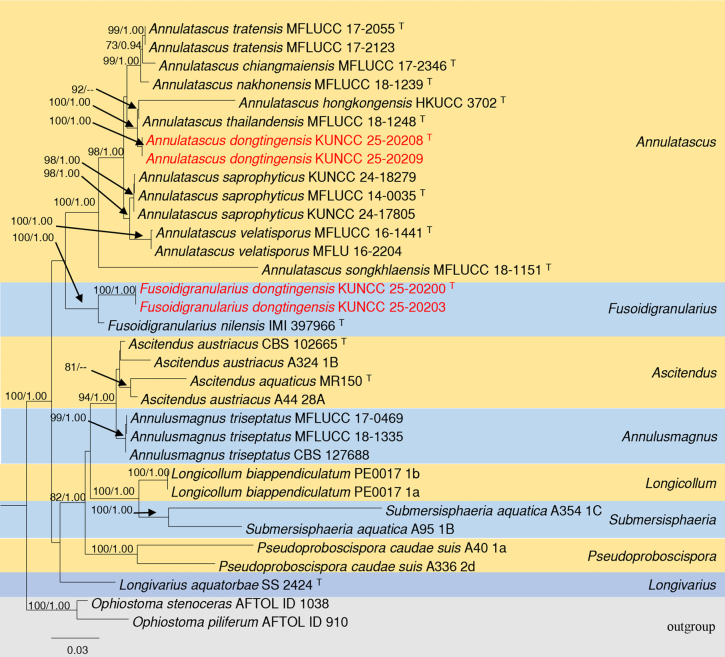
RAxML tree based on a combined dataset of ITS, LSU, and SSU sequence analyses. Bootstrap support values for ML equal to or greater than 70% and Bayesian posterior probabilities (BYPP) equal to or higher than 0.90 are indicated above the nodes as ML/BYPP. The tree is rooted to *Ophiostoma piliferum* (AFTOL ID 910) and *O. stenoceras* (AFTOL ID 1038). Ex-type strains are denoted with T, and the newly generated sequence is indicated in red.

The dataset was analyzed by maximum likelihood (ML) analysis using RAxML-HPC2 v.8.2.12 on the CIPRES Science Gateway web server (Miller et al. 2015). The options ‘Estimate proportion of invariable sites (GTRGAMMA + I)’ and ‘Print branch lengths (−*k*)’ were enabled. The GTRGAMMA model was selected for the bootstrapping phase, and the number of bootstrap iterations was increased from 100 to 1,000. All other parameters remained at their default settings. The model test was conducted using MrMTgui (Nylander 2004). For the multi-locus analysis, appropriate substitution models were assigned to each partition of the concatenated sequences and specified in the command block. Bayesian analysis was conducted using MrBayes version 3.1.2 (Swedish Museum of Natural History, Sweden) to obtain Bayesian posterior probabilities (BYPP) (Huelsenbeck and Ronquist 2001). Two parallel Metropolis-coupled Markov chain Monte Carlo chains were run for up to 2,000,000 generations, with sampling every 100^th^ generation. The analysis was automatically halted once the average standard deviation of split frequencies dropped below 0.01. The distribution of log-likelihood scores was examined to determine the stationary phase for each search and to decide whether extra runs were required to achieve convergence, using the program Tracer 1.5 (University of Edinburgh, UK) (Rambaut et al. 2007). The first 10% of generated trees were discarded, and the remaining 90% of trees were used to calculate posterior probabilities of the majority-rule consensus tree. Bayesian posterior probability (BYPP) values greater than 0.90 are given above each node (Fig. 1). The phylogenetic tree was viewed in FigTree v.1.4.4 (University of Edinburgh, UK) (Rambaut 2018) and edited in Adobe Photoshop CS6 (Adobe Systems, USA).

## Results

### Phylogenetic analyses

The combined ITS, LSU, and SSU sequence dataset contains 32 strains, including the newly generated sequences and outgroup taxa. The final alignment contains 2,496 total characters, including gaps (LSU: 1–866 bp, ITS: 867–1492 bp, SSU: 1493–2496 bp). The best-scoring RAxML tree achieved a final ML optimization likelihood value of –9570.015782. The model produced 712 distinct alignment patterns and 36.30% undetermined characters or gaps. Estimated base frequencies are listed as follows: A = 0.250397, C = 0.241989, G = 0.286811, T = 0.220803, substitution rates AC = 1.110390, AG = 1.783066, AT = 1.054505, CG = 1.074020, CT = 4.744397, GT = 1.000000, and gamma distribution shape parameter alpha = 0.711547. Phylogenetic analyses based on ML and BI methods produced trees with topologies largely consistent with those reported in previous studies (Dong et al. 2021; Yang et al. 2023).

### Taxonomy

#### Annulatascus dongtingensis

Taxon classificationFungiAnnulatascalesAnnulatascaceae

Z.Y. Wang, K.D. Hyde & Q. Zhao sp. nov.

585B00DD-DB4D-5802-889C-EB1645D04FD8

Index Fungorum: IF905205

Facesoffungi Number: FoF19657

Fig. 2

##### Etymology.

The epithet “*dongtingensis*” refers to the collecting site where the holotype was collected.

**Figure 2.**
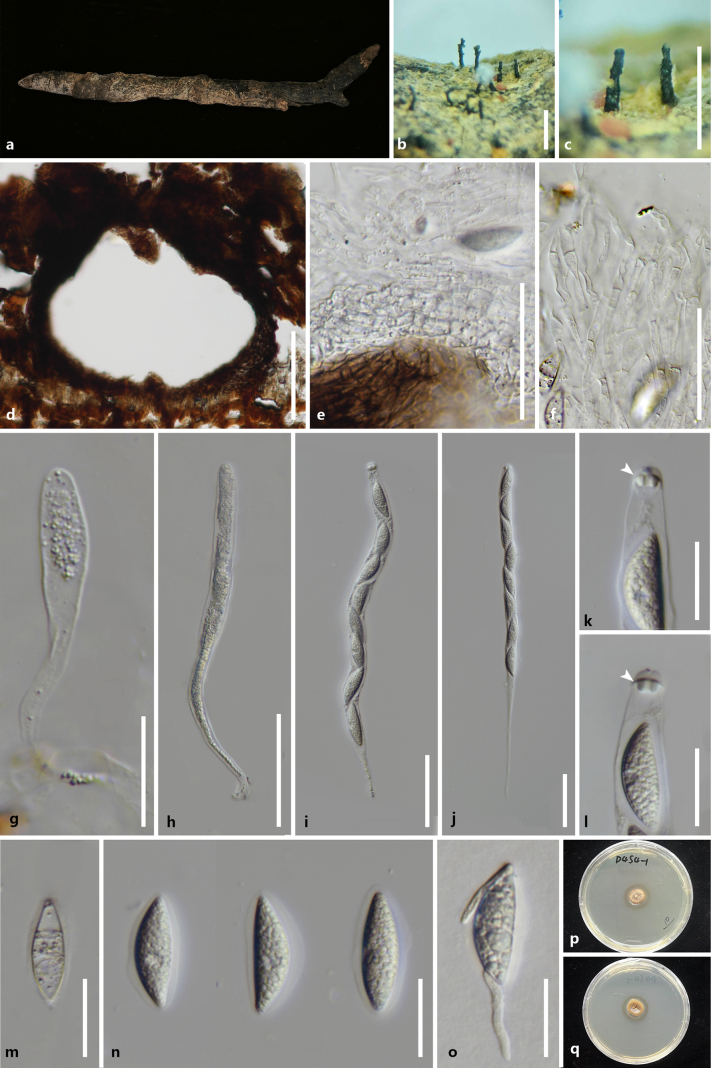
*Annulatascus dongtingensis* (HKAS 150455, holotype). **a–c**. Ascomata on natural substrate; **d**. Section of an ascoma; **e**. Peridium; **f**. Paraphyses; **g–j**. Asci; **k, l**. Apical ring (white arrows); **m, n**. Ascospores; **o**. Germinated ascospore; **p**. Culture on PDA from above; **q**. Culture on PDA from below. Scale bars: 1 cm (**b, c**); 100 μm (**d**); 50 μm (**e, f, h–j**); 20 μm (**g, k–o**).

##### Holotype.

HKAS150455.

##### Description.

***Saprobic*** on submerged wood. **Sexual morph: *Ascomata*** 200–350 µm high × 210–360 µm diam. (x̄ = 270 × 275 µm, *n* = 10), scattered to gregarious, immersed with long neck erumpent straight through the host substrate surface, black, coriaceous, subglobose to ellipsoidal. ***Ostiole*** 400–750 µm (x̄ = 520 µm, *n* = 10) long, central, black, subcylindrical or conical, curved or straight, periphysate. ***Peridium*** 14–30 µm wide (x̄ = 23.3 µm, *n* = 20), with a brown to dark brown outer layer composed of ***textura prismatica*** to ***textura angularis***, intermingled with host tissues, inner layer composed of hyaline ***textura angularis*** cells. ***Paraphyses*** 3–6 µm wide × 160–220 µm length (x̄ = 4.4 × 185 µm, *n* = 30), hyaline, numerous, unbranched, septate, constricted or not at the septa. ***Asci*** 220–270 × 11–14 µm (x̄ = 252 × 12 µm, *n* = 10), 8-spored, unitunicate, cylindrical, apex rounded, with an up to 120 μm long or rarely short pedicel, and a large, wedge-shaped, apical ring, 3–4.2 μm high × 5.5–6.3 μm wide (x̄ = 3.7 × 5.9 µm, *n* = 10). ***Ascospores*** 29–32 × 9–10 µm (x̄ = 30.6 × 9.5 µm, *n* = 30), hyaline, aseptate, fusiform, straight to slightly curved, minutely guttulate, thin-walled, smooth-walled, with a mucilaginous sheath. **Asexual morph**: Undetermined.

##### Culture characteristics.

Ascospore germinate on PDA within 24 h at 25 °C in a dark incubator. Germ tubes are produced from both ends. Colonies reach 2 cm diam after one month at 25 °C, circular, cottony surface, umbonate, entire edge, dense hyphae, with brown sparse mycelium ring, light brown to yellowish-white from above, yellow-brown around the brown center from below, no pigment produced.

##### Material examined.

China • Hunan Province, Yuanjiang City, Dongting Lake (28°47'58.08"N, 112°36'19.09"E, 11 m), submerged wood in shoreline of Dongting Lake, 21 December 2024, Zhi-Yang Wang, D4S4-1 (HKAS 150455, holotype); ex-type KUNCC 25-20208; *ibid*., D4S4-1A (HKAS 150456, isotype), living culture KUNCC 25-20209.

##### GenBank numbers.

KUNCC 25-20208 = ITS: PZ174008, LSU: PZ174012, SSU: PZ174016; KUNCC 25-20209 = ITS: PZ174009, LSU: PZ174013, SSU: PZ174017.

##### Notes.

Based on a combined phylogenetic analysis of LSU, ITS, and SSU sequence data, *Annulatascus dongtingensis* forms a well-supported clade sister to *A. hongkongensis* and *A. thailandensis* (Fig. 1). Among these species, *A. dongtingensis* can be readily distinguished from *A. hongkongensis* by its narrower asci (252 × 12 µm, *L*/*W* ≈ 21 vs. 257 × 26 µm, *L*/*W* ≈ 9.9), smaller ascospores (30.6 × 9.5 µm, *L*/*W* ≈ 3.2 vs. 36 × 14 µm, *L*/*W* ≈ 2.6), and number of septa per ascospore (aseptate vs. 3-septate) (Ho et al. 1999). In addition, the paraphyses of *A. dongtingensis* are distinctly narrower (3–6 µm vs. 6.5–8.5 µm) (Ho et al. 1999). *Annulatascus dongtingensis* also differs from *A. thailandensis* in having smaller asci (252 × 12 µm, *L*/*W* ≈ 21 vs. 310 × 13.7 µm, *L*/*W* ≈ 22.6) and narrower ascospores (30.6 × 9.5 µm, *L*/*W* ≈ 2.6 vs. 30.8 × 11.2 µm, *L*/*W* ≈ 2.8) (Hyde et al. 2020). Further, *A. dongtingensis* possesses aseptate ascospores, whereas *A. thailandensis* has 1–2-septate ascospores (Hyde et al. 2020). The new species is also morphologically distinct in its immersed ascomata with a straight neck, in contrast to the superficial ascomata of *A. thailandensis*, which bear long or short necks that lie horizontally or obliquely across the substrate surface (Zhang et al. 2017; Hyde et al. 2020; Dong et al. 2021). The base pair comparisons (excluding gaps) of LSU show that *A. dongtingensis* differs from *A. hongkongensis* and *A. thailandensis* by 5.4% (45/827 bp) and 0.5% (4/827 bp), respectively. However, low sequence divergence is also common within the genus; for example, *A. thailandensis* differs from *A. saprophyticus* by 0.8% (7/829 bp) and from *A. tratensis* by 0.9% (8/850 bp). The combination of these morphological differences, together with phylogenetic evidence, clearly supports the recognition of *Annulatascus dongtingensis* as a novel species.

#### Fusoidigranularius dongtingensis

Taxon classificationFungiAnnulatascalesAnnulatascaceae

Z.Y. Wang, K.D. Hyde & Q. Zhao sp. nov.

7DEF9862-5373-5380-A432-A9C433393F8C

Index Fungorum: IF905206

Facesoffungi Number: FoF19658

Fig. 3

##### Etymology.

The epithet “*dongtingensis*” refers to the collecting site where the fungus was collected.

**Figure 3.**
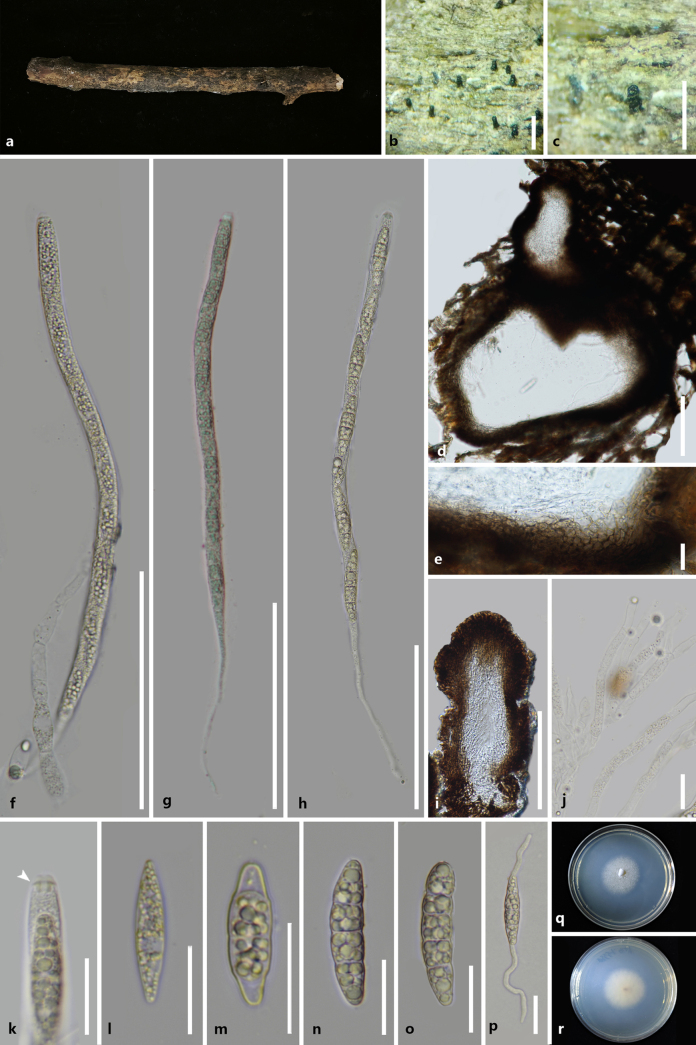
*Fusoidigranularius dongtingensis* (HKAS 150454, holotype). **a–c**. Ascomata on natural substrate; **d**. Section of an ascoma; **e**. Peridium; **f–h**. Asci; **i**. Section of ascomatal neck; **j**. Paraphyses; **k**. Apical ring (white arrows); **l–o**. Ascospores; **p**. Germinated ascospore; **q**. Culture on PDA from above; **r**. Culture on PDA from below. Scale bars: 500 μm (**b, c**); 100 μm (**d, f–i**); 20 μm (**e, j–o**).

##### Holotype.

HKAS 150454.

##### Description.

***Saprobic*** on submerged wood. **Sexual morph: *Ascomata*** 240–410 µm high × 210–400 µm diam. (x̄ = 321 × 271 µm, *n* = 10), scattered to gregarious, immersed, ostiolate, black, coriaceous, subglobose to obpyriform. ***Ostiole*** 130–200 µm long × 80–110 µm wide (x̄ = 168 × 93 µm, *n* = 10), central, black, subcylindrical, straight, periphysate. ***Peridium*** 22–70 µm wide (x̄ = 42 µm, *n* = 30) with 6–11 layers, a dark brown outer part composed cell of ***textura angularis***, inner part composed of 2–4 layers, pale brown to hyaline ***textura angularis*** to ***textura prismatica*** cells. ***Paraphyses*** 3.5–8.4 µm wide × 150–250 µm length (x̄ = 5.7 × 196 µm, *n* = 30), hyaline, numerous, unbranched or rarely branched, septate, constricted or not at the septa, tapering distally. ***Asci*** 306–413 × 8–10 µm (x̄ = 360 × 9 µm, *n* = 10), 8-spored, unitunicate, cylindrical, apex rounded, with up to 200 μm long or rarely short pedicel and a large, refractive, wedge-shaped, apical ring, 2.6–3.7 μm high × 4.5–5.4 μm wide (x̄ = 3.1 × 4.9 µm, *n* = 10). ***Ascospores*** 33–40 × 7.5–9 µm (x̄ = 36.5 × 8.5 µm, *n* = 30), hyaline, 3-septate or occasionally 4-septate, constricted at the septa, fusoid, both ends rounded, straight to slightly curved, guttulate, smooth-walled. **Asexual morph**: Undetermined.

##### Culture characteristics.

Ascospore germinate on PDA within 24 h at 25 °C in a dark incubator. Germ tubes are produced from both ends. Colonies reach 3.4 cm diam after one month at 25 °C, circular, rough surface, umbonate, filiform edge, dense hyphae, white from above, yellow-white around brown spot in the center from below, no pigment.

##### Material examined.

China • Hunan Province, Yiyang City, Dongting Lake (28°53'58.58"N, 112°20'14.88"E, 10 m), submerged wood in shoreline of Dongting Lake, 20 December 2024, D3S3-1 (HKAS 150454, holotype); ex-type KUNCC 25-20200; *ibid*., D3S5-1 (HKAS150457, isotype), living culture KUNCC 25-20203.

##### GenBank numbers.

KUNCC 25-20200 = ITS: PZ174006, LSU: PZ174010, SSU: PZ174014; KUNCC 25-20203 = ITS: PZ174007, LSU: PZ174011, SSU: PZ174015.

##### Notes.

In the phylogenetic analysis, *Fusoidigranularius dongtingensis* clustered with *F. nilensis* (Fig. 1). Although morphologically similar, the new species can be distinguished from *F. nilensis* by several characters. *Fusoidigranularius dongtingensis* has smaller ostioles (130–200 × 80–110 µm vs. 240–360 × 96–112 µm), narrower asci (360 × 9 µm, *L*/*W* ≈ 40.0 vs. 322.5 × 12.2 µm, *L*/*W* ≈ 26.4), and fewer septa per ascospore [3(–4)-septate vs. 5–9(–11)-septate] (Abdel-Wahab et al. 2011). In addition, the position and orientation of the ostiolar neck differ between the two species: in *F. dongtingensis*, the neck is straight and projects vertically through the host substrate, whereas in *F. nilensis*, it is oriented horizontally and lies along the substrate surface (Abdel-Wahab et al. 2011; Zhang et al. 2017; Dong et al. 2021). The base pair comparisons (excluding gaps) of LSU show that *F. dongtingensis* differs from *F. nilensis* by 1.98% (16/807 bp). Therefore, *Fusoidigranularius dongtingensis* is introduced as a novel species based on morphological and phylogenetic analyses.

## Discussion

Freshwater fungi represent an important yet still incompletely documented component of global fungal diversity (Calabon et al. 2023). Recent studies have identified over 3,870 freshwater species, which is minuscule compared to the estimated 1.5 million fungal species (Hawksworth 1991; Dai et al. 2015; Calabon et al. 2022). Recent studies have indicated that the number of freshwater fungal taxa has increased rapidly due to intensified exploration, particularly in Asia, where more than 200 novel species were described after 2015 from China and Thailand alone (Bao et al. 2021; Calabon et al. 2021; Dong et al. 2021; Xu et al. 2025). Despite this progress, global freshwater fungal diversity remains underestimated, with many regions and habitats still poorly explored (Calabon et al. 2022; Calabon et al. 2023).

In China, freshwater fungal research has expanded considerably over the past decades, with studies conducted in regions such as Hong Kong, Yunnan, and other southwestern provinces, contributing numerous new taxa and records (Cai et al. 2002; Luo et al. 2004; Bao et al. 2018, 2021; Xu et al. 2025). However, compared with tropical hotspots such as Southeast Asia, many freshwater ecosystems in China, especially large river basins such as the Yangtze, are still poorly sampled. In addition, previous studies have mainly focused on a limited range of substrates, especially submerged wood, whereas other habitats, such as water, foam, and rocks, are still less explored (Shivarov et al. 2017; Raposeiro et al. 2018; Hosoya et al. 2019).

Another major challenge in freshwater fungal research is the absence of molecular data (Calabon et al. 2023). Many taxa, including those in *Annulatascaceae* and related groups, were originally described based solely on morphology, and numerous species still lack sequence data, such as *Annulatascus apiculatus*, *A. citrisporus*, and *A. fusiformis*, resulting in unresolved phylogenetic placements (Zhang et al. 2017; Réblová et al. 2018; Hyde et al. 2020; Dong et al. 2021). For instance, large-scale phylogenetic studies of freshwater *Sordariomycetes* have demonstrated that *Annulatascaceae* is polyphyletic, with members distributed across multiple clades (Zhang et al. 2017; Luo et al. 2019). This led to the introduction of six new families, including *Atractosporaceae*, *Barbatosphaeriaceae*, *Conlariaceae*, *Lentomitellaceae*, *Pseudoproboscisporaceae*, and *Woswasiaceae*, to accommodate the taxa that morphologically belong to *Annulatascaceae* (Zhang et al. 2017; Dong et al. 2021). These findings highlight the need for integrative taxonomic approaches combining morphology and multi-locus phylogeny.

The present study contributes to addressing these gaps by documenting two new freshwater taxa from Dongting Lake, a major freshwater system within the Yangtze River basin. Phylogenetically, *Annulatascus dongtingensis* forms a well-supported lineage closely related to tropical species such as *A. hongkongensis* and *A. thailandensis*, suggesting that members of this genus are more widely distributed across climatic zones than previously recognized (Ho et al. 1999; Hyde et al. 2020; Dong et al. 2021). This finding provides an example of how increased sampling in underexplored regions can reveal hidden diversity and expand known biogeographic ranges.

The second species, *Fusoidigranularius dongtingensis*, further contributes to the understanding of evolutionary patterns within *Annulatascaceae*-like taxa. The genus *Fusoidigranularius* was established based on phylogenetic evidence to accommodate species previously misclassified in *Annulatascus* (Abdel-Wahab et al. 2011; Dong et al. 2021). It also reveals that certain morphological traits, such as ostiolar neck or ascomata orientation, can vary among closely related taxa, pointing to evolutionary plasticity of these characters (Zhang et al. 2017; Dong et al. 2021). Notably, horizontally oriented ascomata, which are also the key trait of *Fusoidigranularius*, are the defining feature of two *Annulatascaceae*-like families, *Atractosporaceae* and *Pseudoproboscisporaceae*, further underscoring such plasticity (Zhang et al. 2017; Hyde et al. 2020; Dong et al. 2021). However, such differences may be influenced by environmental conditions, as morphological plasticity has been widely documented in freshwater fungi. For instance, features such as ascomatal position and neck orientation may vary in response to ecological factors, including water flow, substrate structure, and oxygen availability (Shearer et al. 2007; Hyde et al. 2016; Calabon et al. 2022). In the present study, this character is interpreted with caution and in conjunction with phylogenetic evidence and other morphological traits (Zhang et al. 2017; Dong et al. 2021). The distinct phylogenetic placement of *F. dongtingensis*, together with differences in morphology, supports its recognition as a separate species. This highlights the importance of integrating morphological and molecular data when evaluating taxonomically informative characters in freshwater fungi, particularly in groups where morphological convergence and environmental plasticity are common. However, given the very low LSU sequence divergence (0.5%, 4/827 bp) between *Annulatascus thailandensis* and *A. dongtingensis*, which is comparable to or even lower than some accepted intraspecific divergences within the genus, it is tentatively suggested that these two taxa may be conspecific. If synonymized, the resulting taxon would be morphologically almost indistinguishable from *A. hongkongensis* in terms of ascus and ascospore dimensions. Nevertheless, the observed morphological differences, including ascospore septation and ascomatal orientation, should not be disregarded. Therefore, further studies, including re-collection of *A. hongkongensis* and multi-locus phylogenetic analyses, are strongly recommended to clarify the taxonomic relationships among these taxa.

Overall, the findings reinforce the view that *Annulatascaceae* and allied taxa remain taxonomically challenging due to polyphyly, morphological homoplasy, and incomplete sampling. At the same time, they show that continued exploration of freshwater habitats, especially in under-sampled regions like central China, can greatly improve the understanding of fungal diversity and evolution.

Future studies should focus on expanding sampling efforts across diverse freshwater habitats and substrates, including underexplored ecological niches such as members of *Nelumbonaceae* and soils under hot springs (Shivarov et al. 2017; Raposeiro et al. 2018; Hosoya et al. 2019). In addition, the integration of high-throughput sequencing and multi-locus phylogenetic analyses will be crucial for resolving species boundaries and uncovering cryptic diversity (Calabon et al. 2023). Increasing the availability of sequence data from type specimens and establishing more culture collections will also be essential to stabilize taxonomy and improve the classification of freshwater fungi.

## Supplementary Material

XML Treatment for Annulatascus dongtingensis

XML Treatment for Fusoidigranularius dongtingensis

## Additional information

### Artificial Intelligence (AI) use

The authors accept full responsibility for the content of the manuscript, including the disclosure of any use of AI.

No AI tools were used in the preparation of this manuscript.

### Author ORCIDs

Z.-Y. Wang https://orcid.org/0009-0006-6770-5189

V. Thiyagaraja https://orcid.org/0000-0002-8091-4579

S.-C. He https://orcid.org/0009-0008-7364-4727

N. Kularathnage https://orcid.org/0000-0001-6623-5791

L.-L. Yuan https://orcid.org/0009-0006-0570-6960

R. Cheewangkoon https://orcid.org/0000-0001-8576-3696

K.D. Hyde https://orcid.org/0000-0002-2191-0762

Q. Zhao https://orcid.org/0000-0001-8169-0573

### Data availability

All of the data that support the findings of this study are available in the main text.

## References

[B1] Abdel-Wahab MA, Abdel-Aziz FA, Mohamed SS, Abdel-Aziz AE (2011) *Annulatascus nilensis* sp. nov., a new freshwater ascomycete from the River Nile, Egypt. IMA Fungus 2: 1–6. 10.5598/imafungus.2011.02.01.01 PMC3317367 22679581

[B2] Bao DF, Luo ZL, Liu JK, Bhat DJ, Sarunya N, Li WL, Su HY, Hyde KD (2018) Lignicolous freshwater fungi in China III: Three new species and a new record of *Kirschsteiniothelia* from northwestern Yunnan Province. Mycosphere 9: 755–768. 10.5943/mycosphere/9/4/4

[B3] Bao DF, Hyde KD, McKenzie EHC, Jeewon R, Su H-Y, Nalumpang S, Luo Z-L (2021) Biodiversity of lignicolous freshwater hyphomycetes from China and Thailand and description of sixteen species. Journal of Fungi 7: 669. 10.3390/jof7080669 PMC8399276 34436208

[B4] Boonyuen N, Sri-indrasutdhi V, Suetrong S, Sivichai S, Jones EB (2012) *Annulatascus aquatorba* sp. nov., a lignicolous freshwater ascomycete from Sirindhorn Peat Swamp Forest, Narathiwat, Thailand. Mycologia 104: 746–757. 10.3852/11-238 22223172

[B5] Cai L, Tsui C, Zhang KQ, Hyde K (2002) Aquatic fungi from Lake Fuxian, Yunnan, China. Fungal Diversity 9: 57–70.

[B6] Calabon MS, Jones EBG, Boonmee S, Doilom M, Lumyong S, Hyde KD (2021) Five novel freshwater ascomycetes indicate high undiscovered diversity in lotic habitats in Thailand. Journal of Fungi 7: 117. 10.3390/jof7020117 PMC7914987 33562556

[B7] Calabon MS, Hyde KD, Jones EBG, Luo ZL, Dong W, Hurdeal VG, Gentekaki E, Rossi W, Leonardi M, Thiyagaraja V, Lestari AS, Shen HW, Bao DF, Boonyuen N, Zeng M (2022) Freshwater fungal numbers. Fungal Diversity 114: 3–235. 10.1007/s13225-022-00503-2

[B8] Calabon MS, Hyde KD, Jones EBG, Bao DF, Bhunjun CS, Phukhamsakda C, Shen HW, Gentekaki E, Al Sharie AH, Barros J, Chandrasiri KSU, Hu DM, Hurdeal VG, Rossi W, Valle LG, Zhang H, Figueroa M, Raja HA, Seena S, Song HY, Dong W, El-Elimat T, Leonardi M, Li Y, Li YJ, Luo ZL, Ritter CD, Strongman DB, Wei MJ, Balasuriya A (2023) Freshwater fungal biology. Mycosphere 14: 195–413. 10.5943/mycosphere/14/1/4

[B9] Campbell J, Shearer CA (2004) *Annulusmagnus* and *Ascitendus*, two new genera in the *Annulatascaceae*. Mycologia 96: 822–833. 10.1080/15572536.2005.11832929 21148902

[B10] Dai YC, Cui BK, Si J, He SH, Hyde KD, Yuan HS, Liu XY, Zhou LW (2015) Dynamics of the worldwide number of fungi with emphasis on fungal diversity in China. Mycological Progress 14: 1–9. 10.1007/s11557-015-1084-5

[B11] Dayarathne MC, Maharachchikumbura SSN, Phookamsak R, Fryar SC, To-anun C, Jones EBG, Al-Sadi AM, Zelski SE, Hyde KD (2016) Morpho-molecular characterization and epitypification of *Annulatascus velatisporus*. Mycosphere 7: 1389–1398. 10.5943/mycosphere/7/9/12

[B12] Dong W, Hyde KD, Jeewon R, Doilom M, Yu XD, Wang GN, Liu NG, Hu DM, Nalumpang S, Zhang H (2021) Towards a natural classification of *Annulatascaceae*-like taxa II: introducing five new genera and eighteen new species from freshwater. Mycosphere 12: 1–88. 10.5943/mycosphere/12/1/1

[B13] Hall TA (1999) BioEdit: a user-friendly biological sequence alignment editor and analysis program for Windows 95/98/NT. Oxford, 95–98. 10.1007/978-1-4757-0905-6_31

[B14] Hawksworth DL (1991) The fungal dimension of biodiversity: magnitude, significance, and conservation. Mycological Research 95: 641–655. 10.1016/S0953-7562(09)80810-1

[B15] Ho WH, Ranghoo VM, Hyde KD, Hodgkiss IJ (1999) Ascal ultrastructural study in *Annulatascus hongkongensis* sp. nov., a freshwater ascomycete. Mycologia 91: 885–892. 10.1080/00275514.1999.12061094

[B16] Ho WH, Yanna, Hyde KD, Hodgkiss IJ (2002) Seasonality and sequential occurrence of fungi on wood submerged in Tai Po Kau Forest Stream, Hong Kong. Fungal Diversity 10: 21–43.

[B17] Hosoya T, Tsukaya H, Suleiman M (2019) Survey of stream spora in Maliau River in Borneo. Bulletin of the National Museum of Nature and Science Series B, Botany 45: 577–562.

[B18] Huelsenbeck JP, Ronquist F (2001) MRBAYES: Bayesian inference of phylogenetic trees. Bioinformatics 17: 754–755. 10.1093/bioinformatics/17.8.754 11524383

[B19] Hyde KD (1992) Tropical Australian freshwater fungi. II.* *Annulatascus velatispora* gen. et sp. nov., *A. bipolaris* sp. nov. and *Nais aquatica* sp.nov. (*Ascomycetes*). Australian Systematic Botany 5: 117–124. 10.1071/SB9920117

[B20] Hyde KD, Fryar S, Tian Q, Bahkali AH, Xu J (2016) Lignicolous freshwater fungi along a north–south latitudinal gradient in the Asian/Australian region; can we predict the impact of global warming on biodiversity and function? Fungal Ecology 19: 190–200. 10.1016/j.funeco.2015.07.002

[B21] Hyde KD, Norphanphoun C, Maharachchikumbura SSN, Bhat DJ, Jones EBG, Bundhun D, Chen YJ, Bao DF, Boonmee S, Calabon MS, Chaiwan N, Chethana KWT, Dai DQ, Dayarathne MC, Devadatha B, Dissanayake AJ, Dissanayake LS, Doilom M, Dong W, Fan XL, Goonasekara ID, Hongsanan S, Huang SK, Jayawardena RS, Jeewon R, Karunarathna A, Konta S, Kumar V, Lin CG, Liu JK, Liu NG, Luangsa-ard J, Lumyong S, Luo ZL, Marasinghe DS, McKenzie EHC, Niego AGT, Niranjan M, Perera RH, Phukhamsakda C, Rathnayaka AR, Samarakoon MC, Samarakoon SMBC, Sarma VV, Senanayake IC, Shang QJ, Stadler M, Tibpromma S, Wanasinghe DN, Wei DP, Wijayawardene NN, Xiao YP, Yang J, Zeng XY, Zhang SN, Xiang MM (2020) Refined families of *Sordariomycetes*. Mycosphere 11: 305–1059. 10.5943/mycosphere/11/1/7

[B22] Jayawardena RS, Hyde KD, Chethana KWT, Daranagama DA, Dissanayake AJ, Goonasekara ID, Manawasinghe IS, Mapook A, Jayasiri SC, Karunarathna A, Li CG, Phukhamsakda C, Senanayake IC, Wanasinghe DN, Camporesi E, Bulgakov TS, Li X, Liu M, Zhang W, Yan JY (2018) Mycosphere Notes 102–168: Saprotrophic fungi on *Vitis* in China, Italy, Russia and Thailand. Mycosphere 9: 1–114. 10.5943/mycosphere/9/1/1

[B23] Karimi O, Hyde K, Asghari R, Kandawatte T, Kaewchai S, Alotibi F, Li Q (2025) Peat swamp *Ascomycota* associated with palms (*Arecaceae*) from Narathiwat, Thailand. Mycosphere 16: 2456–2575. 10.5943/mycosphere/16/1/14

[B24] Katoh K, Standley DM (2013) MAFFT multiple sequence alignment software version 7: improvements in performance and usability. Molecular Biology and Evolution 30: 772–780. 10.1093/molbev/mst010 PMC3603318 23329690

[B25] Luo J, Yin JF, Cai L, Zhang KQ, Hyde KD (2004) Freshwater fungi in Lake Dianchi, a heavily polluted lake in Yunnan, China. Fungal Diversity 16: 93–112.

[B26] Luo ZL, Maharachchikumbur SSN, Liu XY, Li SH, Chen LJ, Zhou D, Su HY, Hyde KD (2015) *Annulatascus saprophyticus* sp. nov. and *Pseudoannulatascus* gen. nov. to accommodate *Annulatascus biatriisporus* (*Annulatascales*, *Sordariomycetes*) from Thailand. Phytotaxa 239: 174–182. 10.11646/phytotaxa.239.2.6

[B27] Luo ZL, Hyde KD, Liu JK, Bhat DJ, Bao DF, Li WL, Su HY (2018) Lignicolous freshwater fungi from China II: Novel *Distoseptispora (Distoseptisporaceae)* species from northwestern Yunnan Province and a suggested unified method for studying lignicolous freshwater fungi. Mycosphere 9: 444–461. 10.5943/mycosphere/9/3/2

[B28] Luo ZL, Hyde KD, Liu JK, Maharachchikumbura SSN, Jeewon R, Bao DF, Bhat DJ, Lin CG, Li WL, Yang J, Liu NG, Lu YZ, Jayawardena RS, Li JF, Su HY (2019) Freshwater *Sordariomycetes*. Fungal Diversity 99: 451–660. 10.1007/s13225-019-00438-1

[B29] Maharachchikumbura SSN, Hyde KD, Jones EBG, McKenzie EHC, Huang SK, Abdel-Wahab MA, Daranagama DA, Dayarathne M, D’souza MJ, Goonasekara ID, Hongsanan S, Jayawardena RS, Kirk PM, Konta S, Liu JK, Liu ZY, Norphanphoun C, Pang KL, Perera RH, Senanayake IC, Shang QJ, Shenoy BD, Xiao YP, Bahkali AH, Kang JC, Somrothipol S, Suetrong S, Wen TC, Xu JC (2015) Towards a natural classification and backbone tree for *Sordariomycetes*. Fungal Diversity 72: 199–301. 10.1007/s13225-015-0331-z

[B30] Miller MA, Schwartz T, Pickett BE, He S, Klem EB, Scheuermann RH, Passarotti M, Kaufman S, O’Leary MA (2015) A RESTful API for access to phylogenetic tools via the CIPRES science gateway. Evolutionary Bioinformatics Online 11: 43–48. 10.4137/EBO.S21501 PMC4362911 25861210

[B31] Nylander JAA (2004) MrModeltest v2. Uppsala: Evolutionary Biology Centre. Uppsala University.

[B32] Rambaut A (2018) FigTree–Tree Figure Drawing Tool Version v. 1.4.4. Institute of Evolutionary Biology, University of Edinburgh, Edinburgh.

[B33] Rambaut A, Drummond AJ, Suchard M (2007) Tracer v1.6. http://beast.bio.ed.ac.uk.Tracer

[B34] Raposeiro PM, Faustino H, Ferreira V, Gonçalves V (2018) Aquatic *Hyphomycetes* from streams on Madeira Island (Portugal). Biodiversity Data Journal 8: e53690. 10.3897/BDJ.8.e53690 PMC7363711 32733142

[B35] Réblová M, Miller AN, Réblová K, Štěpánek V (2018) Phylogenetic classification and generic delineation of *Calyptosphaeria* gen. nov., *Lentomitella*, *Spadicoides* and *Torrentispora (Sordariomycetes)*. Studies in Mycology 89: 1–62. 10.1016/j.simyco.2017.11.004 PMC5773705 29367793

[B36] Réblová M, Winka K (2001) Generic concepts and correlations in ascomycetes based on molecular and morphological data: *Lecythothecium duriligni* gen. et sp. nov. with a *Sporidesmium* anamorph, and *Ascolacicola austriaca* sp. nov. Mycologia 93: 478–493. 10.1080/00275514.2001.12063181

[B37] Senanayake IC, Rathnayaka AR, Marasinghe DS, Calabon MS, Gentekaki E, Lee HB, Hurdeal VG, Pem D, Dissanayake LS, Wijesinghe SN, Bundhun D, Nguyen TT, Goonasekara ID, Abeywickrama PD, Bhunjun CS, Jayawardena RS, Wanasinghe DN, Jeewon R, Bhat DJ, Xiang MM (2020) Morphological approaches in studying fungi: collection, examination, isolation, sporulation and preservation. Mycosphere 11: 2678–2754. 10.5943/mycosphere/11/1/20

[B38] Shearer CA, Descals E, Kohlmeyer B, Kohlmeyer J, Marvanová L, Padgett D, Porter D, Raja HA, Schmit JP, Thorton HA, Voglymayr H (2007) Fungal biodiversity in aquatic habitats. Biodiversity and Conservation 16: 49–67. 10.1007/s10531-006-9120-z

[B39] Shearer CA, Raja HA, Miller AN, Nelson P, Tanaka K, Hirayama K, Marvanová L, Hyde KD, Zhang Y (2009) The molecular phylogeny of freshwater *Dothideomycetes*. Studies in mycology 64: 145–153. 10.3114/sim.2009.64.08 PMC2816971 20169028

[B40] Shivarov VV, Thüs H, Denchev CM (2017) First records of two freshwater lichens, *Hydropunctaria scabra* and *Verrucaria alpicola*, from Bulgaria. Mycobiota 7: 1–5. 10.12664/mycobiota.2017.07.01

[B41] Spatafora JW, Sung GH, Johnson D, Hesse C, O’Rourke B, Serdani M, Spotts R, Lutzoni F, Hofstetter V, Miadlikowska J, Reeb V, Gueidan C, Fraker E, Lumbsch T, Lücking R, Schmitt I, Hosaka K, Aptroot A, Roux C, Miller AN, Geiser DM, Hafellner J, Hestmark G, Arnold AE, Büdel B, Rauhut A, Hewitt D, Untereiner WA, Cole MS, Scheidegger C, Schultz M, Sipman H, Schoch CL (2006) A five-gene phylogeny of *Pezizomycotina*. Mycologia 98: 1018–1028. 10.1080/15572536.2006.11832630 17486977

[B42] Thiyagaraja V, He SC, Luo L, Meng QF, Yang HD, Yang YY (2024) Research Profiles-I. The research of Vinodhini Thiyagaraja and her team at Kunming Institute of Botany, CAS, P.R. China. Current Research in Environmental & Applied Mycology. Journal of Fungal Biology 14(1): 49–62. 10.5943/cream/14/1/2

[B43] Tsui CKM, Hyde KD, Hodgkiss IJ (2000) Biodiversity of fungi on submerged wood in Hong Kong streams. Aquatic Microbial Ecology 21: 289–298. 10.3354/ame021289

[B44] Vaidya G, Lohman DJ, Meier R (2011) SequenceMatrix: concatenation software for the fast assembly of multi‐gene datasets with character set and codon information. Cladistics 27: 171–180. 10.1111/j.1096-0031.2010.00329.x 34875773

[B45] Vilgalys R, Hester M (1990) Rapid genetic identification and mapping of enzymatically amplified ribosomal DNA from several *Cryptococcus* species. Journal of Bacteriology 172: 4238–4246. 10.1128/jb.172.8.4238-4246.1990 PMC213247 2376561

[B46] Vu D, Groenewald M, De Vries M, Gehrmann T, Stielow B, Eberhardt U, Al-Hatmi A, Groenewald JZ, Cardinali G, Houbraken J, Boekhout T, Crous PW, Robert V, Verkley GJM (2019) Large-scale generation and analysis of filamentous fungal DNA barcodes boosts coverage for kingdom fungi and reveals thresholds for fungal species and higher taxon delimitation. Studies in mycology 92: 135–154. 10.1016/j.simyco.2018.05.001 PMC6020082 29955203

[B47] White TJ, Bruns T, Lee S, Taylor J (1990) 38 -Amplification and direct sequencing of fungal ribosomal RNA genes for phylogenetics. In: Innis MA, Gelfand DH, Sninsky JJ, White TJ (Eds) PCR Protocols. Academic Press, San Diego, 315–322. 10.1016/B978-0-12-372180-8.50042-1

[B48] Wong SW, Hyde KD, Jones EBG (1998) *Annulatascaceae*, a new ascomycete family from the tropics. Systema Ascomycetum 16: 17–25.

[B49] Xu RJ, Hyde KD, Li JN, Boonmee S, Liu NG, Yang J, Li Y, Bao DF, Shen HW, Zhu XT, Zhu YA, Li TS, Xu K, Yu FM, Lu JR, Lei L, Wu N, Wu DM, Gao N, Jia PS, He XL, Al-Otibi F, Zhou DQ, Liu JK, Lu YZ, Luo ZL, Yang ZL, Zhao Q (2025) Lignicolous freshwater fungi of the pan Qinghai-Xizang Plateau, China. Fungal Diversity 133: 23–234. 10.1007/s13225-025-00555-0

[B50] Yang J, Liu LL, Jones EG, Hyde KD, Liu ZY, Bao DF, Liu NG, Li WL, Shen HW, Yu XD, Liu JK (2023) Freshwater fungi from karst landscapes in China and Thailand. Fungal Diversity 119: 1–212. 10.1007/s13225-023-00514-7

[B51] Zelski SE (2015) A monograph of the freshwater ascomycete family *Annulatascaceae*: a morphological and molecular study. Ph.D., Urbana, Illinois, University of Illinois at Urbana-Champaign.

[B52] Zhang H, Dong W, Hyde KD, Maharachchikumbura SSN, Hongsanan S, Jayarama Bhat D, Al-Sadi AM, Zhang D (2017) Towards a natural classification of *Annulatascaceae*-like taxa: introducing *Atractosporales* ord. nov. and six new families. Fungal Diversity 85: 75–110. 10.1007/s13225-017-0387-z

[B53] Zhang N, Castlebury LA, Miller AN, Huhndorf SM, Schoch CL, Seifert KA, Rossman AY, Rogers JD, Kohlmeyer J, Volkmann-Kohlmeyer B, Sung GH (2006) An overview of the systematics of the *Sordariomycetes* based on a four-gene phylogeny. Mycologia 98: 1076–1087. 10.1080/15572536.2006.11832635 17486982

